# History of falls and fear of falling are predictive of future falls: Outcome of a fall rate model applied to the Swiss CHEF Trial cohort

**DOI:** 10.3389/fragi.2022.1056779

**Published:** 2022-12-14

**Authors:** Christina Wapp, Anne-Gabrielle Mittaz Hager, Roger Hilfiker, Philippe Zysset

**Affiliations:** ^1^ ARTORG Center for Biomedical Engineering Research, University of Bern, Bern, Switzerland; ^2^ HES-SO Valais-Wallis, School of Health Sciences of Physiotherapy, Leukerbad, Switzerland

**Keywords:** older adults, falls, prediction, risk factors, count regression

## Abstract

**Background:** A third of adults aged 65 years and older fall every year, and falls are a common cause of unintentional injuries. Accurate identification of people at risk of falling is an important step in the implementation of preventive strategies.

**Objective:** Our aim was to investigate the association of fall risk factors with number of reported falls in terms of incidence rate ratios and to develop a fall rate prediction model.

**Methods:** In the randomized controlled trial Swiss CHEF, multiple fall risk variables were assessed in community-dwelling older adults at baseline examination, including age, sex, body mass index, fear of falling, number of falls during the prior 12 months, scores on several physical performance tests, comorbidities, and quality of life. Over the following 6 months, interventions were administered in the form of three home-based exercise programs. Participants were subsequently followed up for another 6 months. Falls were reported prospectively using monthly calendars. Incidence rate ratios were derived *via* negative binomial regression models. Variable selection for the prediction model was conducted using backward elimination and the least absolute shrinkage and selection operator method; the model with the smallest prediction error was then identified.

**Results:** Associations with the number of reported falls were found for number of prior falls, fear of falling, balance and gait deficits, and quality of life. The final model was derived *via* backward elimination, and the predictors included were prior number of falls and a measure of fear of falling.

**Outcome:** Number of prior falls and fear of falling can be used as predictors in a personalized fall rate estimate for community-dwelling older adults. Recurrent fallers having experienced four or more falls are especially at risk of falling again.

## 1 Introduction

Approximately one-third of adults aged 65 years and older fall every year (World Health Organization, 2008). At the same time, falls are among the leading causes of unintentional injuries in this age group, resulting in increased morbidity and mortality (Sjögren and Björnstig, 1989; James et al., 2020; Injury Data Visualization Tools, 2022). Accordingly, it seems clear that the prevention of falls is an important topic and one of broad interest (World Health Organization, 2008). However, the identification of those at risk of falling and therefore in need of intervention is an ongoing challenge.

Various risk factors associated with falls have been identified, including older age, female sex, a history of falls, fear of falling, balance and gait deficits, and cognitive impairment (Deandrea et al., 2010). To identify individuals at risk of falling, numerous fall risk assessment tools have been developed. Such tools normally consist of physical performance tests, questionnaires, or self-reported measures. While some evaluate individual risk factors, others integrate multiple factors within the same assessment (Fabre et al., 2010; Lusardi et al., 2017). Up to this point, only a small number of tools have shown sufficient predictive power to successfully discriminate between fallers and non-fallers (Gates et al., 2008; Lusardi et al., 2017).

Most tools produce a classification as to whether an individual is at risk of falling or not (either yes/no, or a probability between 0 and 1), and associations between fall risk factors and number of falls are usually reported in the form of odds ratios. However, since the risk of injury increases proportionally with each additional fall, a model able to predict an expected number of falls would potentially improve the identification of at-risk individuals. A statistical method capable of providing such an estimate is a count regression model (Cameron and Trivedi, 2013; Hilbe, 2014). Ullah *et al.* (2010) showed that count regression is a suitable method for analysis of fall data. Under this approach, the incidence of falls is the dependent variable, and fall risk factors are independent variables. The output takes the form of a baseline incidence of falls, reported along with rate ratios that describe how this baseline incidence changes depending on the value of each risk factor. So far, only a small number of studies have investigated risk of falling in terms of rate ratios (Damián et al., 2013; Gade et al., 2021).

Against this background, the aim of this analysis was to investigate the association of prospectively recorded fall numbers with various fall risk factors, as assessed in the Swiss CHEF cohort, in terms of rate ratios, and to develop a prediction model to estimate an expected fall rate.

## 2 Methods

### 2.1 Reporting guidelines

This manuscript follows the guidelines for Transparent Reporting of a multivariable prediction model for Individual Prognosis Or Diagnosis (TRIPOD) (Collins et al., 2015). The completed checklist is provided in the Supplementary Material.

### 2.2 Study design

The Swiss CHEF Trial is a multi-center randomized controlled trial that was conducted between 2016 and 2022 to compare the effects of three home-based exercise programs on fall prevention. The study was registered with the clinical trials registry ClinicalTrials.gov (https://clinicaltrials.gov/ct2/show/NCT02926105), and the study protocol has been published previously (Mittaz Hager et al., 2019). Briefly, after enrollment in the study, participants underwent a baseline examination in which their demographic characteristics, history of falls, fear of falling, physical performance on several tests, health state, and quality of life were assessed. Subsequently, the participants were divided into three intervention groups with block-randomization and stratification for age, sex, and risk of falling (assessed as part of the inclusion criteria). The three intervention groups were: 1) a group who followed the experimental intervention program of interest, namely the Test&Exercise program; 2) a control group who followed the Otago exercise program; and 3) a second control group who were administered the “Helsana” intervention. Test&Exercise is a training program developed at the Haut école spécialisée de Suisse occidentale (HES-SO) located in Leukerbad, Switzerland. The program consists of 50 physical tasks that are combined to create a personalized training module depending on their perceived difficulty as rated by the participant during test exercises. Otago is a well-known fall prevention program; it consists of 22 exercises whose levels are defined by physiotherapists (Robertson and Campbell, 2003). Finally, the Helsana control intervention represented usual care; this consists of a booklet containing twelve exercise cards and safety advice produced by the Swiss healthcare insurance provider Helsana (Stürze im Alter müssen nicht sein, 2022).

Participants in the Test&Exercise and Otago intervention groups received eight sessions of physiotherapist instruction and four phone calls over the course of 6 months. Those in the Helsana control group received one session of instruction and four phone calls over the same period. Follow-up lasted for 6 months. During the intervention and follow-up periods, incidents of falls were recorded prospectively using monthly fall calendars. After 6 and 12 months, participants were assessed by blinded assessors on the same variables as measured at the baseline examination. To avoid bias, the instructors who administered the intervention programs were not involved in conducting the examinations at baseline, 6, or 12 months. The study was approved by the relevant Swiss Ethics Committees on research involving humans (registration number 2016-00931).

### 2.3 Study participants

Participants included were community-dwelling adults at least 65 years old and classified as at risk of falling (having a history of falls in the previous 12 months or a perceived fear of falling, as measured by a score of at least 20 points on the Falls Efficacy Scale International, or FES-I). Exclusion criteria were severe visual impairment, receipt of physiotherapeutic treatment with balance training, cognitive impairment (<24 points on the Mini Mental State Examination), or contraindication by the referring physician. Participants with a follow-up time of less than 30 days were excluded from this analysis. All participants provided written informed consent.

### 2.4 Sample size

A sample size calculation was conducted to ensure that differences between the intervention groups would be detectable; this calculation is described in the openly accessible study protocol (Mittaz Hager et al., 2019).

### 2.5 Outcome

The dependent variable was defined as the number of fall incidents as prospectively recorded over the course of 12 months during the intervention and follow-up periods using monthly fall calendars. A fall was defined as “an unexpected event in which the participant comes to rest on the ground, floor, or lower level, with or without injury” (Hauer et al., 2006).

### 2.6 Predictors

*Number of prior falls:* Participants were asked how many times they had fallen within the 12 months prior to the baseline examination. The number of falls was recorded.

*General characteristics:* Age, sex, weight, and height were assessed. Body mass index (BMI) was calculated accordingly. Living environment (rural vs. urban) was also included as predictor. Finally, base of support width was assessed by measuring the distance in cm between the two legs when the participant was standing in a normal position.

*Fear of falling:* Participants were asked the question “Are you afraid of falling?” and provided with three response options: “never”, “sometimes”, or “always”. In addition, the FES-I was administered, with possible scores ranging from 16 to 64 points (Yardley et al., 2005).

*Physical performance tests:* The Timed Up and Go (TUG), the Four Stage Balance Test (FSBT), the Functional Reach Test (FRT), the Five Times Sit-To-Stand test (FTSTS), and a measure of gait speed were administered as tests of participants’ physical performance. TUG measures the time taken to stand up from a chair, walk 3 m, turn around, walk back, and sit down again (Podsiadlo and Richardson, 1991). The FSBT is a balance test with four difficulty levels (1: feet side-by-side, 2: semi-tandem stance, 3: full tandem stance, 4: standing on one foot) (Centers for Disease Control and Prevention, 2021). A level is completed if the participant can hold the position for 10 s without moving the feet or requiring support. The FRT measure the distance a participant can lean forward in a standing position without bending the knees, raising the heels, or taking a step forward (Omaña et al., 2021). The FTSTS measures the time required to stand up five times in a row form a sitting position with crossed arms. Finally, gait speed was measured *via* a 6-m-walk test. All test instruments are described in detail in the published study protocol (Mittaz Hager et al., 2019).

*Health state:* Participants were asked whether they suffered from urinary incontinence, vision impairment, hearing impairment, central neurological disease, or musculoskeletal discomfort. Those who suffered from musculoskeletal discomfort and perceived pain were asked to classify the intensity of their pain with a number between 1 and 100.

*Quality of life:* Quality of life was measured using the Older People’s Quality of Life Questionnaire (OPQOL-35), which consists of 35 questions covering eight domains (Bowling and Stenner, 2011). Possible scores range from 35 to 175.

*Offset and confounding factors:* Study center and intervention group were treated as confounding factors and adjusted for in all univariable models. An offset measured in years to account for differences in follow-up time was included in all the models.

### 2.7 Statistical analysis

#### 2.7.1 Processing of predictors

Age was centered around 70 years and BMI around a value of 25. FES-I and OPQOL-35 scores were shifted to a range with a minimum possible score of 0 by subtraction of 16 and 35 points, respectively. Number of prior falls was entered into the analyses in three forms: as a dichotomized variable representing the presence or absence of prior falls; as a continuous variable; and as a categorical variable with levels 0, 1, 2, 3, 4, and ≥5. For the FSBT, scores of 0 and one were aggregated into a single category due to a low number of observations of a score of 0. Scores on the FTSTS test were transformed into the form used in the Short Physical Performance Battery (Guralnik et al., 1995).

#### 2.7.2 Missing data

We conducted a complete case analysis but report the number of missing observations.

#### 2.7.3 Model fit and variable selection

Incidence rate ratios (IRRs) were derived *via* negative binomial regression models. Univariable models were fit for all candidate predictors and adjusted for observation time, study center, and intervention group. In development of the predictive model, variables were selected using two different methods: backward elimination and the least absolute shrinkage and selection operator (LASSO) method (Tibshirani, 1996; Heinze et al., 2018). The stopping rule for backward elimination was the Bayesian information criterion. For variable selection under the LASSO method, each level of each categorical variable was treated as dummy variable, and the tuning parameter lambda was chosen by selecting the value associated with the smallest mean absolute error in a leave-one-out cross-validation analysis. No variables were forced to remain in the model. The two resulting models derived *via* these two variable selection methods were compared, and the model with the smaller root mean squared prediction error (RMSE) and mean absolute prediction error (MAE) was selected as the final model. Model stability for the backward elimination model was analyzed following the suggestions by Heinze et al. (2018). Briefly, variable selection was repeated 1,000 times using bootstrapped sample data sets. The frequency of inclusion of each candidate predictor was then derived by counting how many times it was included in the selected model across the bootstrapped sample data sets.

#### 2.7.4 Software

Statistical analysis was conducted using R version 4.1.2 with the packages MASS, stats, base, and mpath (R Core Team, 2022; Venables et al., 2002; Wang, 2022; Wang et al., 2015).

## 3 Results

**FIGURE 1:**
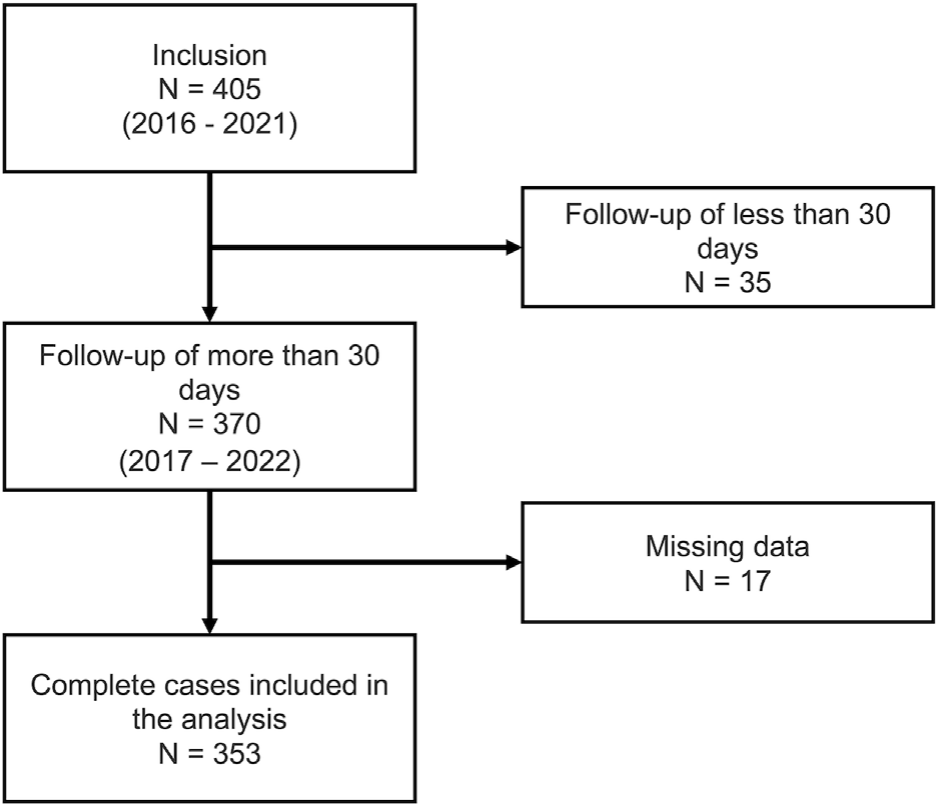
Flow of participants.

In total, 405 participants were enrolled in the Swiss CHEF Trial between 2016 and 2021. Of these, 35 participants dropped out within less than 30 days of the start of the follow-up period. Of the remaining 370 participants, 17 (4.9%) had missing data and were excluded from the analysis. The flow of participants through the study is shown in Figure 1.

The majority of participants (235, 66.6%) were followed up over the course of 12 months; however, 59 (16.7%) dropped out during the intervention (i.e., during the first 6 months) and another 59 (16.7%) dropped out during follow-up (i.e., during the second 6 months). The median age of participants at the start of the study was 79 years, and the majority were female (72.8%). Most were living in a rural environment (80.7%). The median BMI was 25.10 kg/m^2^. In terms of fear of falling, 75 (21.3%) reported not being afraid of falling, 244 (69.1%) reported sometimes being afraid, and 34 (9.6%) reported always being afraid. The median FES-I score was 26 points. The median time for the TUG was 11.6 s, and the median distance measured on the FRT was 25.50 cm. The most common score on the FSBT was two points (138, 39.1%) and the most common score on the FTSTS was one point (126, 35.7%). The median gait speed was 1.07 m/s and median base of support width 29.00 cm. The majority of participants had no hearing problems (204, 57.8%) and did not suffer from urinary incontinence (241, 68.3%) or any neurological disorder (296, 88.5%). However, the majority were affected by vision impairment (291, 82.4%) and reported perceiving musculoskeletal discomfort (305, 85.4%). Around half required a walking aid (168, 47.6%). The median pain score was 45 and the median OPQOL-35 score was 139. A detailed summary of all baseline characteristics and prospectively reported falls during follow-up is presented in Table 1.

**TABLE 1:** Baseline characteristics of the Swiss CHEF Trial cohort.

Variable	Level and measure	Value	NAs
**General characteristics**			
Age (years)	Median [IQR]	79 [73, 84]	-
Sex	Female, n (%)	257 (72.8)	-
	Male, n (%)	96 (27.2)	
Body mass index (kg/m^2^)	Median [IQR]	25.10 [22.28, 28.09]	4
Living area	Urban, n (%)	68 (19.3)	-
	Rural, n (%)	285 (80.7)	
Participation time in months	Median [IQR]	12.00 [8.00, 12.00]	-
Intervention group	Control Helsana, n (%)	68 (19.3)	-
	Otago, n (%)	138 (39.1)	
	Test&Exercise, n (%)	147 (41.6)	
**Falls and fear of falling**			
Prior fall number	Mean	1.47	-
	Median [IQR]	1 [0, 2]	
	Range	0–30	
	0 falls, n (%)	113 (32.0)	
	1 fall, n (%)	124 (35.1)	
	2 falls, n (%)	62 (17.6)	
	3 falls, n (%)	25 (7.1)	
	4 falls, n (%)	11 (3.1)	
	≥5 falls, n (%)	18 (5.1)	
Incident falls	Mean	1.05	-
	Median [IQR]	0 [0, 1]	
	Range	0–20	
	0 falls, n (%)	204 (57.8)	
	1 fall, n (%)	69 (19.6)	
	2 falls, n (%)	38 (10.8)	
	3 falls, n (%)	20 (5.7)	
	4 falls, n (%)	8 (2.3)	
	≥5 falls, n (%)	14 (4.0)	
FES-I score	Median [IQR]	26 [21, 32]	-
Fear of falling	Never, n (%)	75 (21.3)	-
	Sometimes, n (%)	244 (69.1)	
	Always, (%)	34 (9.6)	
**Physical performance tests**			
Timed Up and Go	Median [IQR]	11.61 [9.27, 14.38]	3
Functional Reach Test	Median [IQR]	25.50 [18.93, 31.23]	-
Four Stage Balance Test	Median [IQR]	3 [2, 4]	-
	Score 0, n (%)	5 (1.4)	
	Score 1, n (%)	23 (6.5)	
	Score 2, n (%)	138 (39.1)	
	Score 3, n (%)	107 (30.3)	
	Score 4, n (%)	80 (22.7)	
Gait speed (m/s)	Median [IQR]	1.07 [0.82, 1.32]	-
Five Times Sit-To-Stand	Median time [IQR]	15.29 [12.34, 19.13]	41*
	Median score [IQR]	2 [1, 3]	-
	Score 0, n (%)	37 (10.5)	
	Score 1, n (%)	126 (35.7)	
	Score 2, n (%)	74 (21.0)	
	Score 3, n (%)	69 (19.6)	
	Score 4, n (%)	47 (13.3)	
Base of support width (cm)	Median [IQR]	29.00 [26.45, 32.10]	-
**Health state and comorbidities**			
Hearing problems	No, n (%)	204 (57.8)	3
	Yes, n (%)	149 (42.2)	
Vision impairment	No, n (%)	62 (17.6)	4
	Yes, n (%)	291 (82.4)	
Walking aid	No, n (%)	185 (52.4)	1
	Yes, n (%)	168 (47.6)	
Urinary incontinence	No, n (%)	241 (68.3)	4
	Yes, n (%)	112 (31.7)	
Musculoskeletal disorder	No, n (%)	48 (13.6)	3
	Yes, n (%)	305 (86.4)	
Neurological disorder	No, n (%)	296 (83.9)	3
	Yes, n (%)	57 (16.2)	
Pain (range 0–100)	Median [IQR]	45 [12, 60]	-
**Quality of life**			
OPQOL-35	Median [IQR]	139 [129, 152]	1

* 41 participants were not able to perform the test, resulting in a score of 0 points. Abbreviations: NAs, not available; n = number; % = percentage; IQR, interquartile range; OPQOL-35, Older People’s Quality of Life Questionnaire.

In total, 369 falls were registered during intervention and follow-up; 149 (42.2%) participants reported at least one fall, and 80 (22.7%) fell multiple times (Table 1). For the 12 months prior to the baseline assessment, participants reported 517 falls in total: 240 (68.0%) reported having fallen at least once during this period, whereas 116 (32.9%) had fallen multiple times.

IRRs for all candidate predictors are presented in Table 2. Associations with number of prospectively reported falls were found for the following variables: enrollment in the Otago intervention program compared to the control intervention (IRR: 2.25, 95% CI 1.28-3.95); number of prior falls operationalized as a dichotomized variable (IRR: 1.71, 95% CI 1.11-2.62) and as a continuous variable (IRR: 1.26, 95% CI 1.18-1.34); having experienced four falls (IRR: 3.15, 95% CI 1.28-7.70) or ≥5 falls (IRR: 7.20, 95% CI 3.62-14.31) on the measure of number of prior falls as a categorical variable; FES-I score (IRR: 1.05, 95% CI 1.03-1.07); reporting “always” experiencing fear of falling as compared to “never” (IRR: 3.77, 95% CI 1.83-7.80); TUG time (IRR: 1.05, 95% CI 1.01-1.08); a score of 0 or 1 on the FSBT compared to a score of 4 (IRR: 3.05, 95% CI 1.53- 6.08); base of support width (IRR: 1.05, 95% CI 1.01-1.09); and OPQOL-35 score (IRR: 0.98, 95% CI 0.97-0.99). All other predictors were not found to be associated with prospectively recorded number of falls.

**TABLE 2:** Incidence rate ratios derived *via* negative binomial regression models.

Variable and level for categorical variables		Incidence rate ratio (95% CI)
**General characteristics**		
Age		0.99 (0.96, 1.02)
Sex	Male	*ref*
	Female	0.70 (0.46, 1.07)
Body mass index		1.01 (0.97, 1.05)
Living environment	Rural	*ref*
	Urban	3.34 (0.27, 41.10)
Intervention	Control Helsana	*ref*
	Otago	2.25 (1.28, 3.95)
	Test&Exercise	1.63 (0.92, 2.87)
**Falls and fear of falling**		
Prior falls (binary)	no	*ref*
	no	1.71 (1.11, 2.62)
Prior fall number (continuous)		1.26 (1.18, 1.34)
Prior fall number (categorical)	0 falls	*ref*
	1 fall	1.04 (0.66, 1.62)
	2 falls	1.10 (0.64, 1.89)
	3 falls	1.89 (0.95, 3.73)
	4 falls	3.15 (1.28, 7.70)
	≥5 falls	7.20 (3.62, 14.31)
FES-I score		1.05 (1.03, 1.07)
Fear of falling	Never	*ref*
	Sometimes	1.54 (0.92, 2.56)
	Always	3.77 (1.83, 7.80)
**Physical performance tests**		
Timed Up and Go		1.05 (1.01, 1.08)
Functional Reach Test		0.99 (0.97, 1.01)
Four Stage Balance Test	Score 4	*ref*
		Score 3	0.88 (0.53, 1.46)
		Score 2	0.82 (0.51, 1.34)
		Score 0 or 1	3.05 (1.53, 6.08)
	Gait speed		0.60 (0.32, 1.10)
	Five Times Sit-To-Stand	Score 4	*ref*
		Score 3	1.39 (0.60, 2.82)
		Score 2	1.88 (0.94, 3.73)
		Score 1	1.25 (0.65, 2.39)
		Score 0	1.28 (0.56, 2.89)
	Base of support width		1.05 (1.01, 1.09)
	**Health state and comorbidities**		
	Hearing problems	No	*ref*
		Yes	0.76 (0.51, 1.12)
	Vision problems	No	*ref*
		yes	1.05 (0.62, 1.77)
	Walking aid	No	*ref*
		Yes	1.43 (0.97, 2.11)
	Urinary incontinence	No	*ref*
		Yes	1.08 (0.71, 1.63)
	Musculoskeletal disorder	No	*ref*
		Yes	0.77 (0.44, 1.33)
	Neurological disorder	No	*ref*
		Yes	1.13 (0.67, 1.89)
	Pain	0–25 points	*ref*
		26–50 points	0.98 (0.62, 1.55)
		51–75 points	0.62 (0.37, 1.04)
		76–100 points	1.84 (0.91, 3.72)
	**Quality of life**		
	OPQOL-35		0.98 (0.97, 0.99)

All univariable models were adjusted for study center and intervention group.

Abbreviations: ref = reference group; OPQOL-35 , Older People’s Quality of Life Questionnaire.

**FIGURE 2:**
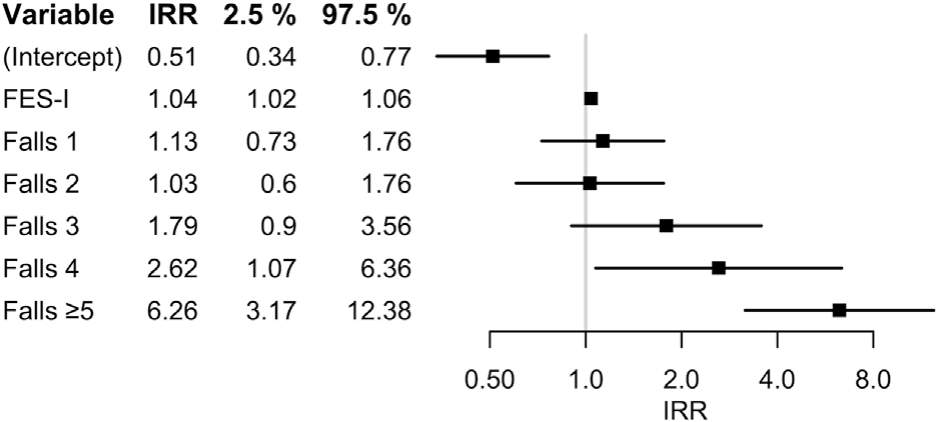
Incidence rate ratios (IRR) and corresponding 95% confidence intervals in the model generated with variable selection via backward elimination, in numerical form (left) and as forest plot (right).

Variable selection with backward elimination resulted in a model including prior number of falls and FES-I score as the only predictor variables. The coefficient estimates together with prediction errors are presented in Table 3, and the corresponding rate ratios, in numerical form and as a forest plot, are presented in Figure 2. The RMSE and MAE were 2.02 and 1.15, respectively; internal cross-validation increased these values to 2.17 and 1.21, respectively. LASSO variable selection resulted in a model including having experienced five or more prior falls and a score of 0 or one points on the FSBT as predictors. Model coefficients and detailed prediction errors for the LASSO model are presented in Supplementary Table S1. Both apparent error and cross-validated error were higher for the LASSO model compared to the backward elimination model.

**TABLE 3:** Coefficients, bootstrap inclusion frequency, and predictive performance for the backward elimination model.

Final prediction model with backward elimination		
Variable	Coefficient estimates (95% CI) on log scale	Bootstrap inclusion frequency (%)
Intercept	−0.67 (−1.08, −0.27)	100
Prior fall number = [1]	0.12 (−0.32, 0.57)	80.8
Prior fall number = [2]	0.03 (−0.50, 0.56)	80.8
Prior fall number = [3]	0.58 (−0.10, 1.27)	80.8
Prior fall number = [4]	0.96 (0.07, 1.85)	80.8
Prior fall number = [5+]	1.83 (1.15, 2.52)	80.8
FES-I	0.04 (0.02, 0.06)	74.2
theta	0.71 (0.46, 0.95)	
Predictive performance		
Measure	Value (IQR)	
RMSE	2.02 (0.43, 0.72, 1.24)	
MAE	1.15	
CV RMSE	2.17 (0.51, 0.72, 1.17)	
CV MAE	1.21	

Abbreviations: CI, confidence interval; FES-I, falls efficacy scale international; RMSE, root mean squared error; MAE, mean absolute error; IQR, interquartile range with median; CV, cross-validated.

Model stability investigation for the backward elimination model resulted in a bootstrap inclusion frequency of 80.8% for prior number of falls and 74.2% for FES-I score (Table 3). All other candidate predictors had an inclusion frequency of less than 50%; the complete list can be found in Supplementary Table S3. An example of how to use the model to calculate an individual’s expected fall rate can also be found in the Supplementary Material S1.

## 4 Discussion

The aim of this study was to investigate the associations between various fall risk factors and number of prospectively reported falls, and to develop a personalized fall rate prediction model. This analysis made use of data from the randomized controlled trial Swiss CHEF, which investigated the effects of two different interventions for fall prevention compared to a control intervention in community-dwelling older adults. Candidate predictors included in the analysis were assessed prior to the start of the intervention. Rate ratios were derived *via* negative binomial regression models, and variable selection for the prediction model were conducted using backward elimination and LASSO. The final prediction model was selected on the basis of smallest prediction error. We followed the TRIPOD reporting guidelines for the development of a multivariable prediction model.

The associations observed between risk factors analyzed and prospectively reported number of falls are comparable to other results reported in the literature (Deandrea et al., 2010; Ambrose et al., 2013; Lusardi et al., 2017). A history of falls, gait and balance deficits, fear of falling, and a decrease in quality of life are well-known risk factors for falls in community-dwelling older adults. The strongest associated factor in this analysis was found to be having experienced four or ≥5 prior falls, indicating that individuals with a history of multiple falls are at risk of falling multiple times again. While a single fall might occur at random, recurrent fallers are likely to suffer from persistent deficits that result in an inability to avoid falls.

A large decrease in the total number of falls could be observed in a comparison of the falls reported during the 12 months prior to baseline assessment and during the 12 months of intervention and follow-up. It is plausible that the intervention programs, as well as sensitization to the risk of falling as a result of participation in the study, were the cause of this decrease. Accordingly, this may potentially have resulted in underestimation of the derived incidence rate ratios in comparison to a randomly selected population. An observational study with no intervention program would be required to overcome this issue.

Number of prior falls and FES-I score (as a measure for fear of falling) were the only two predictors included in the final model generated by the variable selection algorithm. There are many reasons why it may not be possible to prevent a particular fall, and a broad combination of factors can plausibly function as causes of a fall. Hence, prior occurrences of falls might be the best reflection of whether factors causing falls are present in a given individual or not. Although other predictors investigated in this analysis were associated with prospectively reported number of falls, each of these variables measured only a single aspect of the risk of falling. Given this fact, the inclusion of prior number of falls in the model immediately produced superior predictive power for the number of future falls compared to the inclusion of other variables. The second predictor included in the model, fear of falling, is known to increase with experience of falls (Fabre et al., 2010). Therefore, an explanation for its predictive power might be the fact that it functions as an alternative measure of the presence of prior falls. Surprisingly, inclusion of the variable representing intervention group did not appear to improve predictive power, although the incidence of falls differed among the intervention groups, as we saw in the univariable analysis.

PREFALL, a fall rate model that was developed using the LASSO method in a recently published study, includes two variables similar to those included in our model, namely the presence of a history of falls (yes/no) and self-perceived risk of falling (Gade et al., 2021). In a comparison of prediction error, PREFALL is superior to our model. Although the apparent prediction error of the model presented here was comparable to that of PREFALL, the cross-validated error was higher, indicating some bias. An error of more than one fall can have substantial influence when screening for people at risk of falling, introducing the potential to miss individuals who are in need of preventive measures. Thus, the identification of further risk predictors resulting in a more accurate prediction is required.

A strength of this study is that the outcome variable, namely the number of falls reported during intervention and follow-up, was recorded prospectively. Prospective recording is known to be more precise compared to retrospective assessment of number of falls (Garcia et al., 2015). In addition, this analysis provided insight into the form in which history of falls is best included as a predictor variable. While use of the information in dichotomized form (yes *versus* no) can only produce changes in the prediction for fallers *versus* non-fallers, use of a continuous variable enables the prediction to be adjusted according to the number of prior falls experienced. However, by introducing the number of prior falls in the form of a categorical variable, we were further able to show that prospective fall rate does not merely increase in a log-linear relationship with increasing number of prior falls, as assumed for a continuous variable; rather, the relationship is a stronger one.

Most fall risk assessment tools evaluate the risk of falling *via* binary logistic regression to identify who is predicted to fall. We believe that analyzing fall count data with rate ratios, as suggested by Ullah et al. (2010), is a better approach. The insight gained as to the probability that someone will fall at all is different from a prediction of how many times someone is expected to fall.

The analysis presented here also has several limitations. First, the Swiss CHEF cohort study was designed as an intervention study. An observational study would have been a superior design for the development of a prediction model. Second, the fall prevalence in this cohort was higher than the prevalence reported in the literature: e.g., in Switzerland the prevalence is reported to be around 25% (Die funktionale Gesundheit von älteren Menschen in Privathaushalten, 2014). This is a consequence of the inclusion criteria, which required participants to be at risk of falling, resulting in a sample population with a higher prevalence of falls compared to a random sample population. Third, the imbalanced nature of the cohort in terms of sex, with a large proportion of women compared to men, is not optimal for such an analysis, since risk factors for falls can differ between sexes. Finally, although count regression models can adjust for differences in observation time *via* an offset variable, shorter observation times can result in both under- and overestimation of an individual’s true number of falls compared to follow-up observation as planned. Further studies are required to overcome those issues, validate the model, and improve its prediction accuracy.

In summary, this analysis showed that history of falls, in terms of prior number of falls, and FES-I score are relevant variables in the prediction of future fall rates. Methodologically, the inclusion of the number of prior falls as a categorical variable has the potential to improve the predictive accuracy of fall risk and fall rate estimation models.

## Data Availability

The raw data supporting the conclusion of this article will be made available by the authors, without undue reservation.

## Abbreviations

BMI, Body mass index; FES-I, Falls Efficacy Scale International; FRT, Functional Reach Test; FTSTS, Five Times Sit-To-Stand; LASSO, Least Absolute Shrinkage and Selection Operator; TUG, Timed Up and Go; OPQOL-35, Older People’s Quality of Life Questionnaire.
