# Corrigendum: History of falls and fear of falling are predictive of future falls: Outcome of a fall rate model applied to the Swiss CHEF trial cohort

**DOI:** 10.3389/fragi.2023.1235431

**Published:** 2023-07-06

**Authors:** Christina Wapp, Anne-Gabrielle Mittaz Hager, Roger Hilfiker, Philippe Zysset

**Affiliations:** ^1^ ARTORG Center for Biomedical Engineering Research, University of Bern, Bern, Switzerland; ^2^ HES-SO Valais-Wallis, School of Health Sciences of Physiotherapy, Leukerbad, Switzerland

**Keywords:** older adults, falls, prediction, risk factors, count regression

In the published article, there was an error in Table 3 as published. The root mean squared error (RMSE) and its interquartile range (IQR) stated as 0.93 (0.58, 0.92, 1.06) is wrong. The corrected numbers are 2.02 (0.43, 0.72, 1.24). The mean absolute error (MAE) stated for the backward elimination model states as 0.84 is wrong. The corrected number is 1.15. The corrected Table 3 and its caption “Coefficients, bootstrap inclusion frequency, and predictive performance for the backward elimination model.” appear below.

**TABLE 3:** Coefficients, bootstrap inclusion frequency, and predictive performance for the backward elimination model.

Final prediction model with backward elimination		
Variable	Coefficient estimates (95% CI) on log scale	Bootstrap inclusion frequency (%)
Intercept	−0.67 (−1.08, −0.27)	100
Prior fall number = [1]	0.12 (−0.32, 0.57)	80.8
Prior fall number = [2]	0.03 (−0.50, 0.56)	80.8
Prior fall number = [3]	0.58 (−0.10, 1.27)	80.8
Prior fall number = [4]	0.96 (0.07, 1.85)	80.8
Prior fall number = [5+]	1.83 (1.15, 2.52)	80.8
FES-I	0.04 (0.02, 0.06)	74.2
theta	0.71 (0.46, 0.95)	
Predictive performance		
Measure	Value (IQR)	
RMSE	2.02 (0.43, 0.72, 1.24)	
MAE	1.15	
CV RMSE	2.17 (0.51, 0.72, 1.17)	
CV MAE	1.21	

Abbreviations: CI, confidence interval; FES-I, falls efficacy scale international; RMSE, root mean squared error; MAE, mean absolute error; IQR, interquartile range with median; CV, cross-validated.

Accoringly, a correction has been made to **Results**, paragraph 5. This sentence previously stated:

“The RMSE and MAE were 0.93 and 0.84, respectively; internal cross-validation increased these values to 2.17 and 1.21, respectively.”

The corrected sentence appears below:

“The RMSE and MAE were 2.02 and 1.15, respectively; internal cross-validation increased these values to 2.17 and 1.21, respectively.”

The authors apologize for this error and state that this does not change the scientific conclusions of the article in any way. The original article has been updated.

