# Supplementary material for: History of falls and fear of falling are predictive of future falls: Outcome of a fall rate model applied to the Swiss CHEF Trial cohort
Source: Front Aging. 2022 Dec 14;3:1056779. doi: 10.3389/fragi.2022.1056779 (PMC9795055; doi:10.3389/fragi.2022.1056779)
Supplement: Supplementary file 1 [file DataSheet1.docx]

Supplementary Material

# Supplementary Tables

Supplementary Table 1. Coefficients and predictive performance measures for the LASSO selected model.

| *LASSO model* | |
| --- | --- |
| Variable | Coefficient estimates (log-scale) |
| Intercept | -2.44 |
| Prior fall number = [≥5) | 0.60 |
| Four stage balance test = [0, 1] | 0.08 |
| *Predictive performance* | |
| Measure | Value |
| RMSE | 2.43 |
| MAE | 1.06 |
| CV RMSE | 2.76 |
| CV MAE | 2.19 |
| *Note:* LASSO = least absolute shrinkage and selection operator, RMSE = rooted mean squared error, MAE = mean absolute error, CV = cross-validated. | |

Supplementary Table 2. Completed list of bootstrap inclusion frequency for model selection with backward elimination.

| Variable | Bootstrap inclusion frequency (%) |
| --- | --- |
| Intercept | 100 |
| Prior fall number | 80.8 |
| FES-I | 74.2 |
| Four stage balance test | 42.3 |
| OPQOL-35 | 31.4 |
| Pain | 30.4 |
| Five times sit to stand | 23.4 |
| Musculoskeletal problems | 21.4 |
| Age | 18.5 |
| Timed Up and Go | 15.7 |
| Functional reach test | 12.0 |
| Sex | 11.8 |
| Gait speed 6 meter | 11.4 |
| randomisation | 10.6 |
| Fear of falling | 9.0 |
| Urinary incontinence | 8.8 |
| Hearing problems | 8.8 |
| Urban/rural | 8.1 |
| Neurological disease | 7.9 |
| Body mass index | 7.1 |
| Walking aid | 7.0 |
| Study center | 6.6 |
| Base support width | 6.4 |
| Vision impairment | 6.2 |
| *Note:* FES-I = Falls Efficacy Scale International, OPQOL-35 = Older People’s Quality of Life Questionnaire | |

# TRIPOD Checklist.

| **Section/Topic** | **Item** | **Checklist Item** | **Page** |
| --- | --- | --- | --- |
| **Title and abstract** | | | |
| Title | 1 | Identify the study as developing and/or validating a multivariable prediction model, the target population, and the outcome to be predicted. | 1 |
| Abstract | 2 | Provide a summary of objectives, study design, setting, participants, sample size, predictors, outcome, statistical analysis, results, and conclusions. | 1, 2 |
| **Introduction** | | | |
| Background and objectives | 3a | Explain the medical context (including whether diagnostic or prognostic) and rationale for developing or validating the multivariable prediction model, including references to existing models. | 2 |
|  | 3b | Specify the objectives, including whether the study describes the development or validation of the model or both. | 2 |
| **Methods** | | | |
| Source of data | 4a | Describe the study design or source of data (e.g., randomized trial, cohort, or registry data), separately for the development and validation data sets, if applicable. | 2 |
|  | 4b | Specify the key study dates, including start of accrual; end of accrual; and, if applicable, end of follow-up. | 2 |
| Participants | 5a | Specify key elements of the study setting (e.g., primary care, secondary care, general population) including number and location of centres. | 2 |
|  | 5b | Describe eligibility criteria for participants. | 3 |
|  | 5c | Give details of treatments received, if relevant. | 2, 3 |
| Outcome | 6a | Clearly define the outcome that is predicted by the prediction model, including how and when assessed. | 3 |
|  | 6b | Report any actions to blind assessment of the outcome to be predicted. | 3 |
| Predictors | 7a | Clearly define all predictors used in developing or validating the multivariable prediction model, including how and when they were measured. | 3 |
|  | 7b | Report any actions to blind assessment of predictors for the outcome and other predictors. | - |
| Sample size | 8 | Explain how the study size was arrived at. | 3 |
| Missing data | 9 | Describe how missing data were handled (e.g., complete-case analysis, single imputation, multiple imputation) with details of any imputation method. | 4 |
| Statistical analysis methods | 10a | Describe how predictors were handled in the analyses. | 4 |
|  | 10b | Specify type of model, all model-building procedures (including any predictor selection), and method for internal validation. | 4 |
|  | 10d | Specify all measures used to assess model performance and, if relevant, to compare multiple models. | 4 |
| Risk groups | 11 | Provide details on how risk groups were created, if done. | - |
| **Results** | | | |
| Participants | 13a | Describe the flow of participants through the study, including the number of participants with and without the outcome and, if applicable, a summary of the follow-up time. A diagram may be helpful. | 4 |
|  | 13b | Describe the characteristics of the participants (basic demographics, clinical features, available predictors), including the number of participants with missing data for predictors and outcome. | 6, 7 |
| Model development | 14a | Specify the number of participants and outcome events in each analysis. | 4, 5, 6 |
|  | 14b | If done, report the unadjusted association between each candidate predictor and outcome. | 8, 9 |
| Model specification | 15a | Present the full prediction model to allow predictions for individuals (i.e., all regression coefficients, and model intercept or baseline survival at a given time point). | 9 |
|  | 15b | Explain how to the use the prediction model. | SM* |
| Model performance | 16 | Report performance measures (with CIs) for the prediction model. | 10 |
| **Discussion** | | | |
| Limitations | 18 | Discuss any limitations of the study (such as nonrepresentative sample, few events per predictor, missing data). | 9, 10 |
| Interpretation | 19b | Give an overall interpretation of the results, considering objectives, limitations, and results from similar studies, and other relevant evidence. | 5, 9 |
| Implications | 20 | Discuss the potential clinical use of the model and implications for future research. | 9, 10 |
| **Other information** | | | |
| Supplementary information | 21 | Provide information about the availability of supplementary resources, such as study protocol, Web calculator, and data sets. | 10 |
| Funding | 22 | Give the source of funding and the role of the funders for the present study. | 10 |

SM = Supplementary Materials

# Example of how to use the prediction model.

The model writes as

$$\log\left( fall rate \right)=-0.67+0.12\cdot I_{Falls=1}+0.03\cdot I_{Falls=2}+0.58\cdot I_{Falls=3}+0.96\cdot I_{Falls=4}+1.83\cdot I_{Falls=5}+0.04\cdot(FESI-16)$$

where $I$ is the indicator function given as

$$I_{condition}=\left\{ \begin{aligned} 1, &condition is true \\ 0, &otherwise \end{aligned} \right.$$

The expected fall rate of a person having reached a FES-I score of 24 with 4 falls experienced in the prior 12 months to examination results in

$$\log\left( fall rate \right)=-0.67+0.96\cdot1+0.04\left( 24-16 \right)=0.61$$

$$fall rate=\exp\left( 0.61 \right)=1.84$$
